# A link between the steepest descent method and fixed-point iterations

**DOI:** 10.1007/s11590-022-01867-9

**Published:** 2022-03-18

**Authors:** Pascal Heid

**Affiliations:** Mathematical Institute, University of Oxford, Woodstock Road, Oxford, OX2 6GG UK

**Keywords:** Fixed-point iterations, Steepest descent method, Preconditioned conjugate gradient method, Preconditioning operator, Sobolev gradient

## Abstract

We will make a link between the steepest descent method for an unconstrained minimisation problem and fixed-point iterations for its Euler–Lagrange equation. In this context, we shall rediscover the preconditioned algebraic conjugate gradient method for the discretised problem. The benefit of the connection of those concepts will be illustrated by a numerical experiment.

## Introduction

Throughout this work, let *X* be a Hilbert space endowed with an inner-product denoted by $(\cdot ,\cdot )_X$ and induced norm $\left\| \cdot \right\| _X$. Furthermore, we consider a functional $\mathsf {H}:X \rightarrow \mathbb {R}$ and are interested in the optimisation problem

1 $$\begin{aligned} {{\,\mathrm{arg\,min}\,}}_{u \in X} \mathsf {H}(u). \end{aligned}$$

In general, there may exist several (local) minimisers or possibly none at all. In this work, we shall make the following assumptions on the functional $\mathsf {H}$:

1. $\mathsf {H}$ is Gateaux-differentiable;
2. $\mathsf {H}$ is strictly convex;
3. $\mathsf {H}$ is weakly coercive, i.e., $\mathsf {H}(u) \rightarrow \infty$ as $\left\| u\right\| _X \rightarrow \infty$.

Those assumptions (H1)–(H3) imply that $\mathsf {H}$ has a unique minimiser $u^\star \in X$; see, e.g., [57, Thm. 25.E]. A well-known procedure to approximate a minimiser of such a functional $\mathsf {H}$ is the *steepest descent method*, introduced by Augustin Cauchy in the work [7]. The main idea of this method is very intuitive: at each iteration step, we move in direction of the steepest descent. In particular, if $u^n \in X$ is a given iterate, then we set

2 $$\begin{aligned} u^{n+1}:=u^n - \delta ^n \nabla \mathsf {H}(u^n), \end{aligned}$$

where $\nabla \mathsf {H}(u^n)$, to be specified in Sect. 2, is the gradient of $\mathsf {H}$ at $u^n$ and $\delta ^n >0$ is an appropriate step-size such that $\mathsf {H}(u^{n+1}) \le \mathsf {H}(u^n)$. The optimal choice of the step-size is given by

$$\begin{aligned} \delta ^n:={{\,\mathrm{arg\,min}\,}}_{t \ge 0} \mathsf {H}(u^n-t \nabla \mathsf {H}(u^n)), \end{aligned}$$

which requires the solution of a one-dimensional optimisation problem. In practice, we may often only approximate the optimal step-size. More comments on that issue will be provided in Remark 3.1 below.

It is well-known, see, e.g., [57, Thm. 25.F], that under the assumptions (H1)–(H3) the unconstrained minimisation problem (1) is equivalent to the operator equation

3 $$\begin{aligned} \text {find} \ u \in X \ \text {such that} \quad \mathsf {F}(u)=0 \quad \text {in} \ X^\star , \end{aligned}$$

where $\mathsf {F}:=\mathsf {H}':X \rightarrow X^\star$ is the Gateaux-derivative of the functional $\mathsf {H}$ and $X^\star$ denotes the dual space of *X*; i.e., the set of all continuous linear functionals from *X* to $\mathbb {R}$. There exists a wide variety of fixed-point iterations for the numerical solution of the problem (3) and, as was shown in [18, 19], in many cases they can be interpreted as an iterative local linearisation procedure, which can be obtained by applying a suitable preconditioning operator to the original Eq. (3). In particular, for any given $u \in X$, let $\mathsf {P}[u]:X \rightarrow X^\star$ be a linear and invertible operator. Then, the operator Eq. (3) is equivalent to the fixed-point equation

4 $$\begin{aligned} \text {find} \ u \in X \ \text {such that} \quad u=u-\mathsf {P}[u]^{-1}\mathsf {F}(u). \end{aligned}$$

This, in turn, gives rise to the fixed-point iteration

5 $$\begin{aligned} u^{n+1}:=u^n-\mathsf {P}[u^n]^{-1}\mathsf {F}(u^n), \end{aligned}$$

where $u^0 \in X$ is an initial guess. In practice, we seldom invert $\mathsf {P}[u^n]$ (in the finite-dimensional setting), but rather solve the linear problem

6 $$\begin{aligned} \text {find} \ u^{n+1} \in X \ \text {such that} \quad \mathsf {P}[u^n]u^{n+1}=\mathsf {P}[u^n]u^n-\mathsf {F}(u^n) \qquad \text {in} \ X^\star \end{aligned}$$

by applying an iterative linear solver. However, for simplicity, the algebraic error will be neglected in this work; i.e., we assume that the linear Eq. (6) is solved exactly. Moreover, problem (6) can be stated equivalently as

7 $$\begin{aligned} \text {find} \ u^{n+1} \in X \ \text {such that} \quad a(u^n;u^{n+1},v)=\langle \ell (u^n),v\rangle \quad \text {for all} \ v \in X, \end{aligned}$$

where

$$\begin{aligned} a(u;v,w):=\langle \mathsf {P}[u]v,w\rangle , \qquad u,v,w \in X, \end{aligned}$$

and

$$\begin{aligned} \langle \ell (u),v\rangle :=\langle \mathsf {P}[u]u-\mathsf {F}(u),v\rangle , \qquad u,v \in X; \end{aligned}$$

here, $\langle \cdot ,\cdot \rangle$ denotes the duality pairing in $X^\star \times X$. We note that some prominent iteration schemes, such as the Zarantonello,^1^ Kačanov,^2^ and Newton methods, can be cast into this unified framework, and we refer to [18]. In the following we assume that the bilinear form $a(u;\cdot ,\cdot ):X \times X \rightarrow \mathbb {R}$ is

1. uniformly coercive, i.e., there exists a constant $\alpha >0$ such that
  $$\begin{aligned} a(u;v,v) \ge \alpha \left\| v\right\| _X^2 \quad \text {for all} \ u,v \in X; \end{aligned}$$
2. uniformly bounded, i.e., there exists a constant $\beta >0$ such that
  $$\begin{aligned} a(u;v,w) \le \beta \left\| v\right\| _X \left\| w\right\| _X \quad \text {for all} \ u,v,w \in X; \end{aligned}$$
3. symmetric, i.e., $a(u;v,w)=a(u;w,v)$ for all $u,v,w \in X$.

We note that those assumptions imply, thanks to the Lax–Milgram theorem, that the operator $\mathsf {P}[u]:X \rightarrow X^\star$ is invertible for any $u \in X$ and that the Eq. (7) has a unique solution for each $n=0,1,2,\dotsc$. We further assume that the operator $\mathsf {F}:X \rightarrow X^\star$ is

1. strongly monotone, i.e., there exists a constant $\nu >0$ such that
  $$\begin{aligned} \langle \mathsf {F}(u)-\mathsf {F}(v),u-v\rangle \ge \nu \left\| u-v\right\| ^2_X \quad \text {for all} \ u,v \in X; \end{aligned}$$
2. Lipschitz continuous, i.e., there exists a constant $L_\mathsf {F}>0$ such that
  $$\begin{aligned} \langle \mathsf {F}(u)-\mathsf {F}(v),w\rangle \le L_\mathsf {F}\left\| u-v\right\| _X \left\| w\right\| _X \quad \text {for all} \ u,v,w \in X. \end{aligned}$$

Under those assumptions, the theory on strongly monotone operator equations yields that Eq. (3) has a unique solution $u^\star \in X$; see, e.g., [57, §25.4]. We recall that this solution is as well the unique minimiser of $\mathsf {H}$ in *X*. We further note that the strong monotonicity (F1) of the operator $\mathsf {F}$ implies the strict convexity (H2) of its potential $\mathsf {H}$.

Under suitable assumptions it can be shown that the potential $\mathsf {H}$ decreases along the sequence $\{u^n\}_n$ generated by the unified iteration scheme (7) in the sense that there exists a constant $C_\mathsf {H}>0$ such that

8 $$\begin{aligned} \mathsf {H}(u^n)-\mathsf {H}(u^{n+1}) \ge C_\mathsf {H}\left\| u^n-u^{n+1}\right\| _X^2 \quad \text {for all} \ n=0,1,2,\dotsc ; \end{aligned}$$

see [19, §2.1] for a general discussion of the property (8) and [16, §2.4] for the required assumptions guaranteeing (8) for the Zarantonello, Kačanov, and Newton methods. In particular, given the monotonicity property (8), the update $-\mathsf {P}[u^n]^{-1}\mathsf {F}(u^n)$ from the fixed-point iteration (5) can be considered as a descent direction of the potential $\mathsf {H}$ at the given iterate $u^n \in X$. This indicates that there might be a link between the steepest descent method (2) for the optimisation problem (1) and the fixed-point iteration (5) for the solution of its Euler–Lagrange Eq. (3).

Indeed, this relation of the operator preconditioning in the context of fixed-point iterations and the steepest descent methods for generalised Sobolev gradients is already known in the existing literature; here, we simply refer to the monograph [9], which discusses this link in an extensive way, and will provide further references at a later stage. This connection will be recovered in Sect. 2 of the present manuscript. Subsequently, in Sect. 3, in the context of second order partial differential equations in divergence form, we will further relate this to the preconditioning of the algebraic gradient descent method of the discretised problem – also this link is already known in the existing literature on Sobolev gradient methods, see, e.g. [9, Ch. 8]. In regard of this insight, we will shortly discuss the nonlinear conjugate gradient method in Sect. 3.4. Subsequently, a numerical experiment will be performed in Sect. 4 in order to demonstrate the usefulness of our theoretical observations. Finally, we will round off our work with some conclusions in Sect. 5.

As should be apparent from the discussion above, the present work rarely contains any new insights for itself, but the purpose is to synthesise the relations and perceptions of different concepts in a condensed and well-understandable way, with an application focus on the unified iteration scheme (6) studied in the author’s previous works [16, 18, 19]. Furthermore, we want to highlight the advantages of the different viewpoints and how this could be used to find efficient iteration schemes. Throughout our paper, we will embed our work in the large body of literature, and point to existing results of the different concepts that are connected herein.

## Link between the steepest descent method and the unified iteration scheme

We will now make the link between the steepest descent method (2) and the unified iteration scheme (5) visible. For that purpose, let us first recall the steepest descent method (2), which involves the gradient $\nabla \mathsf {H}(u^n) \in X$ of $\mathsf {H}$ at $u^n \in X$. By definition it holds that, for fixed $u \in X$,

9 $$\begin{aligned} \langle \mathsf {F}(u),v\rangle =\langle \mathsf {H}'(u),v\rangle =:(\nabla \mathsf {H}(u),v)_X \quad \text {for all} \ v \in X. \end{aligned}$$

In particular, the gradient depends on the considered inner-product, which shall be indicated by a subscript in the following; i.e., we write $\nabla _X \mathsf {H}(u)$ for the gradient of $\mathsf {H}$ at $u \in X$ with respect to the inner-product $(\cdot ,\cdot )_X$. Indeed, this follows the Sobolev gradient approach as introduced in a series of papers by Neuberger, see, e.g., [37, 38, 40, 41], further developed with a focus on weighted inner products by Mahavier [27–30], or Nittka and Sauter [42] in the context of discrete algebraic equations, and applied in various situations by Raza, Sial and co-authors [31, 32, 47–53]. Actually, since the pioneering work of Neuberger, the Sobolev gradient methods have been widely studied and applied for numerous problems, meaning that the reference list above is far from complete. We further refer to the monograph [39] for a very nice summary of the works of Neuberger and Mahavier, and many more results on Sobolev gradient flows.

If we denote by $\mathrm {J}_X:X \rightarrow X^\star$ the Riesz isometry with respect to the inner-product $(\cdot ,\cdot )_X$ on *X*, then we have that $\nabla _X \mathsf {H}(u)=\mathrm {J}_X^{-1} \mathsf {H}'(u)=\mathrm {J}_X^{-1} \mathsf {F}(u)$, cf. (9). In turn, the steepest descent method reads as

10 $$\begin{aligned} u^{n+1}=u^n-\delta ^n\mathrm {J}_X^{-1}\mathsf {F}(u^n), \end{aligned}$$

which coincides with the fixed-point iteration (5) for the preconditioning operator $\mathsf {P}[u]={\delta (u)}^{-1}\mathrm {J}_X$, $u \in X$, where the damping function satisfies $\delta (u^n)=\delta ^n$ for $n=0,1,2,\dotsc$. We note that this specific choice of the preconditioning operator gives rise to the Zarantonello iteration; see the original work [56], or the monographs [36, §3.3] and [57, §25.4]. Moreover, given the assumptions (F1)–(F2), the Zarantonello iteration generates a sequence converging to the unique solution $u^\star \in X$ of (3) for a suitable choice of the damping function $\delta :X \rightarrow \mathbb {R}_{>0}$; see, e.g., the proof of [57, Thm. 25.B].

If $a:X \times X \rightarrow \mathbb {R}$ is a symmetric, coercive, and bounded bilinear form on $X \times X$, then $a(\cdot ,\cdot )$ can be considered as an inner-product on *X* whose corresponding norm $\left\| \cdot \right\| _a$ is equivalent to the norm $\left\| \cdot \right\| _X$; i.e., *X* endowed with the inner-product $a(\cdot ,\cdot )$ and norm $\left\| \cdot \right\| _a$ is a Hilbert space as well. We further note that, in turn, the bilinear form $a:X \times X \rightarrow \mathbb {R}$ induces a linear and invertible operator $\mathsf {P}:X \rightarrow X^\star$ defined by

11 $$\begin{aligned} \langle \mathsf {P}u,v\rangle :=a(u,v) \quad \text {for all} \ u,v \in X. \end{aligned}$$

We may then consider the gradient of $\mathsf {H}$ with respect to the inner-product $a(\cdot ,\cdot )$, i.e., for given $u \in X$,

$$\begin{aligned} a(\nabla _a \mathsf {H}(u),v):=\langle \mathsf {H}'(u),v\rangle \quad \text {for all} \ v \in X. \end{aligned}$$

In view of (11) we have that

$$\begin{aligned} \langle \mathsf {P}\nabla _a \mathsf {H}(u),v\rangle =\langle \mathsf {H}'(u),v\rangle =\langle \mathsf {F}(u),v\rangle \quad \text {for all} \ v \in X, \end{aligned}$$

and therefore $\nabla _a \mathsf {H}(u)=\mathsf {P}^{-1} \mathsf {F}(u)$. In this case, the steepest descent method (2) coincides, up to some damping parameter, with the unified iteration scheme (5) for the preconditioner from (11). In particular, we obtain a preconditioning (of the simple iteration) by a change of the inner-product for the steepest descent method; this was already pointed out in [9, Ch. 7.3].

Finally, similarly as was done in [20] in the context of Sobolev gradient flows for the Gross–Pitaevskii equation, we may consider an inner-product that changes with the iteration; we further refer, e.g., to [9, Ch. 7.3(b)] and [39, Ch. 29.5] for variable inner products in the context of Sobolev gradients, and see the references at the end of the section as well. For fixed $u \in X$, let $a_u=a(u;\cdot ,\cdot ):X \times X \rightarrow \mathbb {R}$ be a symmetric, uniformly coercive and bounded bilinear form, cf. (A1)–(A3). Consequently, for any $u \in X$, the operator $\mathsf {P}[u]: X \rightarrow X^\star$ defined by

12 $$\begin{aligned} \langle \mathsf {P}[u]v,w\rangle :=a_u(v,w)=a(u;v,w), \qquad v,w \in X, \end{aligned}$$

is linear and invertible. Then, we can define the gradient of $\mathsf {H}$ at a given element $u \in X$ with respect to the inner-product $a_u(\cdot ,\cdot )$ by

13 $$\begin{aligned} a_u(\nabla _{a_u} \mathsf {H}(u),v):=\langle \mathsf {H}'(u),v\rangle =\langle \mathsf {F}(u),v\rangle \quad \text {for all} \ v \in X; \end{aligned}$$

i.e., we have that $\nabla _{a_u} \mathsf {H}(u)=\mathsf {P}[u]^{-1}\mathsf {F}(u)$. In turn, the steepest descent method is given by

$$\begin{aligned} u^{n+1}=u^n-\delta (u^n)\mathsf {P}[u^n]^{-1}\mathsf {F}(u^n), \end{aligned}$$

which, for $\delta (u^n)\equiv 1$, $n=0,1,2,\dotsc$, matches our unified iteration scheme (5). In particular, we have shown the result below; in this context, we also want to refer to a very close discussion in [9, Ch. 5].

### Proposition 2.1

Let $\mathsf {P}[u]:X \rightarrow X^\star$, for $u \in X$, be a linear and invertible operator which induces a bilinear form $a_u=a(u;\cdot ,\cdot )$ that satisfies (A1)–(A3) (or vice versa), cf. (12). Then, the unified iteration scheme (5) with preconditioner $\mathsf {P}[u]$ coincides with the steepest descent method (2) with the gradient being taken with respect to the (variable) inner-product $a_u(\cdot ,\cdot )$ and constant step-sizes $\delta ^n \equiv 1$, $n=0,1,2,\dotsc$.

### Remark 2.2

There may result some advantages from this relation between the steepest descent method and the unified fixed-point iteration.

1. The step-size function of the (modified) steepest descent method in the context of Proposition 2.1 is simply given by $\delta \equiv 1$, and thus we do not need to employ, e.g., a line search or trusted region method to determine $\delta ^n$, $n=0,1,2,\dotsc$. We note that the preconditioning operator $\mathsf {P}[u]$ may implicitly include a damping parameter $\delta (u)$; however, in many cases, this damping parameter can be prescribed or can easily be chosen adaptively in such a way that the decay property (8) is satisfied in each iteration step.
2. The convergence of fixed-point iterations is well studied in the literature, also in the context of the adaptive interplay with finite element discretisations, see, e.g., [11–14, 16, 19]. By the identification of the unified iteration scheme (5) and the steepest descent method (2) (with constant step-size function $\delta \equiv 1$), those results also apply to the latter.
3. On the other hand, the steepest descent method serves as basis of the superior (nonlinear) conjugate gradient method. Hence, it might be sensible to consider the nonlinear conjugate gradient method in the case that the gradient is taken with respect to a variable inner-product (induced by a preconditioning operator $\mathsf {P}[u]:X \rightarrow X^\star$ from a fixed-point iteration), cf. (13). Indeed, we will show in the next section that this gives rise to the known preconditioned nonlinear conjugate gradient (PNCG) method in the discrete setting.

We emphasise that there is a large body of literature focusing on the Sobolev (gradient) preconditioning and its convergence properties; we refer, e.g., to [2–5, 8, 9, 22–25, 34, 35, 39] for an incomplete list.

## Nonlinear conjugate gradient method for variable inner-products

For the purpose of examining the nonlinear conjugate gradient method in the context of variable inner-products we will consider a model problem, which will now be introduced.

### Model problem

As our model problem, we will consider the following quasilinear second order elliptic partial differential equation:

14 $$\begin{aligned} \begin{aligned} -\nabla \cdot \{\mu (|\nabla u|^2)\nabla u\}&=g \quad&\text {in} \ \Omega , \\ u&=0 \quad&\text {on} \ \partial \Omega , \end{aligned} \end{aligned}$$

where $\Omega \subset \mathbb {R}^d$, $d \in \{2,3\}$, is an open, bounded, and polygonal domain. More precisely, we set

15 $$\begin{aligned} \mathsf {F}(u):=-\nabla \cdot \{\mu (|\nabla u|^2)\nabla u\}-g \end{aligned}$$

and $X:=H_0^1(\Omega )$, which is the Sobolev space of $H^1$-functions with zero trace along the boundary $\partial \Omega$, in (3). Here, for $u,v \in X$, the inner-product and norm on *X* are defined by $(u,v)_X:=(\nabla u,\nabla v)_{L^2(\Omega )}$ and $\left\| u\right\| _X:=\left\| \nabla u\right\| _{L^2(\Omega )}$, respectively. Furthermore, $g \in L^2(\Omega )$, considered as an element in the dual space $H^{-1}(\Omega ):=H_0^1(\Omega )^\star$, is a given source function and the diffusion coefficient $\mu \in C^1([0,\infty ))$ satisfies the monotonicity condition

16 $$\begin{aligned} m_\mu (t-s) \le \mu (t^2)t-\mu (s^2)s \le M_\mu (t-s), \qquad t \ge s \ge 0, \end{aligned}$$

for some constants $M_\mu \ge m_\mu >0$. Given those assumptions, the nonlinear operator $\mathsf {F}:X \rightarrow X^\star$ from (15) satisfies the conditions (F1) and (F2) with $\nu =m_\mu$ and $L_\mathsf {F}=3 M_\mu$; see, e.g., [57, Prop. 25.26]. We note the weak form of our model problem (14):

17 $$\begin{aligned} \text {find} \ u \in X \ \text {such that} \quad \int _\Omega \mu (|\nabla u|^2)\nabla u \cdot \nabla v \,\mathsf {d}\varvec{x}=\int _\Omega gv \,\mathsf {d}\varvec{x}\quad \text {for all} \ v \in X. \end{aligned}$$

It is straightforward to verify that $\mathsf {F}$ is a potential operator with the potential given by

$$\begin{aligned} \mathsf {H}(u):=\int _\Omega \psi (|\nabla u|^2) \,\mathsf {d}\varvec{x}-\int _\Omega gu \,\mathsf {d}\varvec{x}, \qquad u \in X, \end{aligned}$$

where $\psi (s)=\nicefrac {1}{2} \int _0^s \mu (t) \,\mathsf {d}t$ for $s \ge 0$. As $\mathsf {F}$ is strongly monotone it immediately follows that $\mathsf {H}$ is strictly convex. Furthermore, the Cauchy–Schwarz inequality, the Poincaré–Friedrich inequality (with constant denoted by $C_P$), and the assumption (16) imply that

$$\begin{aligned} \mathsf {H}(u) \ge \frac{m_\mu }{2} \left\| u\right\| ^2_{X}-C_P \left\| g\right\| _{L^2(\Omega )}\left\| u\right\| _X, \end{aligned}$$

thus $\mathsf {H}$ is weakly coercive. In particular, (H1)–(H3) are satisfied. If we further assume that $\mu$ is monotonically decreasing, i.e., $\mu '(t) \le 0$ for all $t \ge 0$, then the Zarantonello iteration, the Kačanov scheme, and the damped Newton method all satisfy — for suitable damping functions — assumptions (A1)–(A3) as well as (8); see [16, 18, 19]. In particular, those references yield that following three methods generate a sequence converging to the unique solution of (3), or, equivalently, of (1):

(i) *Zarantonello iteration,* for $\delta _Z \in (0,\nicefrac {2}{3 M_\mu })$:
  18 $$\begin{aligned} u^{n+1}=u^n-\delta _Z \mathrm {J}_X^{-1}\mathsf {F}(u^n) \quad \text {for all} \ n=0,1,2,\dotsc , \end{aligned}$$
  where $\mathrm {J}_X:X \rightarrow X^\star$ denotes, as before, the Riesz isometry with respect to the inner-product $(\cdot ,\cdot )_X$ on *X*;
(ii) *Kačanov iteration:*
  $$\begin{aligned} u^{n+1}=u^{n}-\mathsf {P}[u^n]^{-1}\mathsf {F}(u^n)\quad \text {for all} \ n=0,1,2,\dotsc , \end{aligned}$$
  where $\langle \mathsf {P}[u]v,w\rangle :=\int _\Omega \mu (|\nabla u|^2) \nabla v \cdot \nabla w\,\mathsf {d}\varvec{x}$ for $u,v,w \in X$, or, equivalently,
  $$\begin{aligned} - \nabla \cdot \big \{\mu (\left| \nabla u^n\right| ^2) \nabla {u^{n+1}}\big \}=g \quad \text {for all} \ n=0,1,2,\dotsc ; \end{aligned}$$
(iii) *Newton iteration,* for a damping parameter $0< \delta _{\mathrm {min}} \le \delta _N(u^n) \le \delta _{\mathrm {max}}<\nicefrac {2 m_\mu }{3 M_\mu }$:
  19 $$\begin{aligned} u^{n+1}=u^{n}-\delta _N(u^n) \mathsf {F}'(u^{n})^{-1}\mathsf {F}(u^{n})\quad \text {for all} \ n=0,1,2,\dotsc ; \end{aligned}$$
  here, for $u\in X$, the Gateaux-derivative $\mathsf {F}'(u)$ of $\mathsf {F}$ is given through
  $$\begin{aligned} \langle \mathsf {F}'(u)v,w\rangle= & {} \int _{\Omega } 2 \mu '(|\nabla u|^2)(\nabla u \cdot \nabla v)(\nabla u \cdot \nabla w) \,\mathsf {d}\varvec{x}\\&+ \int _{\Omega } \mu (|\nabla u|^2)\nabla v \cdot \nabla w \,\mathsf {d}\varvec{x}, \quad v,w \in X. \end{aligned}$$

Indeed, if $u^\star$ denotes the unique solution of (3) and the sequence $\{u^n\}_{n}$ was generated by any of the three iteration schemes (i)–(iii) from above, then [16, Thm. 2.1] implies that

20 $$\begin{aligned} \left\| u^{n}-u^\star \right\| _X \le q^n C \left\| u^0-u^\star \right\| _X, \qquad n=1,2,\dotsc , \end{aligned}$$

where $q \in (0,1)$ is a given contraction factor and $C>0$ is some positive constant independent of *n*. Furthermore, the convergence property (20) remains valid in the discrete setting.

### Discretisation of the model problem

Since $X=H_0^1(\Omega )$ is an infinite-dimensional space, we cannot compute the sequence generated by any of the iteration schemes presented before. In order to cast them into a computational framework, we will consider the discretisation by a conforming finite element method. In particular, for a given triangulation $\mathcal {T}_h$ of $\Omega$, the corresponding finite element space is given by

21 $$\begin{aligned} X_h:=\{u \in H_0^1(\Omega ): u|_K \in \mathbb {P}_{p}(K) \ \forall K \in \mathcal {T}_h\}, \end{aligned}$$

where, for fixed $p \in \mathbb {N}$, $\mathbb {P}_{p}(K)$ signifies the space of all polynomials of total degree at most *p* on $K \in \mathcal {T}_h$. Then, the discretisation of the weak problem (17) reads as

22 $$\begin{aligned} \text {find} \ u_h \in X_h \ \text {such that} \quad \int _\Omega \mu (|\nabla u_h|^2)\nabla u_h \cdot \nabla v \,\mathsf {d}\varvec{x}=\int _\Omega g v \,\mathsf {d}\varvec{x}\quad \text {for all} \ v \in X_h. \end{aligned}$$

Furthermore, upon defining

$$\begin{aligned} \mathsf {B}_h(u;v,w):=\int _\Omega \mu (|\nabla u|^2) \nabla v \cdot \nabla w \,\mathsf {d}\varvec{x}, \qquad u,v,w \in X_h, \end{aligned}$$

and

$$\begin{aligned} \langle \ell _h,v\rangle :=\int _\Omega gv\,\mathsf {d}\varvec{x}, \qquad v \in X_h, \end{aligned}$$

the discrete weak problem (22) can be stated equivalently as follows:

23 $$\begin{aligned} \text {find} \ u_h \in X_h \ \text {such that} \quad \mathsf {B}_h(u_h;u_h,v) =\langle \ell _h,v\rangle \quad \text {for all} \ v \in X_h. \end{aligned}$$

We emphasise that, for any $u \in X_h$, $\mathsf {B}_h(u;\cdot ,\cdot ):X_h \times X_h \rightarrow \mathbb {R}$ is a symmetric, uniformly coercive and bounded bilinear form. In particular, we have that

$$\begin{aligned} \mathsf {B}_h(u;v,v) \ge m_\mu \left\| v\right\| _X^2 \quad \text {for all} \ u,v \in X_h \end{aligned}$$

and

$$\begin{aligned} \mathsf {B}_h(u;v,w) \le M_\mu \left\| v\right\| _X \left\| w\right\| _X \quad \text {for all} \ u,v,w \in X_h. \end{aligned}$$

Consequently, thanks to the Lax–Milgram theorem, (23) has a unique solution. Moreover, if we define $\mathsf {F}_h: X_h \rightarrow X_h^\star$ by

24 $$\begin{aligned} \mathsf {F}_h(u):=\mathsf {B}_h(u;u,\cdot )-\ell _h, \qquad u \in X_h, \end{aligned}$$

then we can state (23) in form of an operator equation:

$$\begin{aligned} \text {find} \ u_h \in X_h \ \text {such that} \quad \mathsf {F}_h(u_h)=0 \qquad \text {in} \ X_h^\star . \end{aligned}$$

Now let $\{\xi _i\}_{i=1}^{m_h}$ be a basis of $X_h$, where $m_h \in \mathbb {N}$ denotes the number of degrees of freedom in $X_h$. Consequently, each element $u \in X_h$ can be written in a unique way as a linear combination of those basis vectors; i.e., $u=\sum _{i=1}^{m_h} c_i \xi _i$, where $c_i \in \mathbb {R}$, for $i \in \{1,\dotsc ,m_h\}$, are the coefficients of *u* with respect to the basis $\{\xi _i\}_{i=1}^{m_h}$. Then, the corresponding linear mapping $\Psi :\mathbb {R}^{m_h} \rightarrow X_h$ defined by $\Psi (\mathbf {u}):=\sum _{i=1}^{m_h} c_i \xi _i$, where $\mathbf {u}=(c_1,\dotsc ,c_{m_h})^{T} \in \mathbb {R}^{m_h}$, is one-to-one; here, $^T$ denotes the matrix transposition. By invoking this isomorphism we may consider the discrete weak Eq. (23) as a problem in $\mathbb {R}^{m_h}$:

25 $$\begin{aligned} \text {find} \ \mathbf {u}_h \in \mathbb {R}^{m_h} \ \text {such that} \quad \mathsf {B}_h(\Psi (\mathbf {u}_h);\Psi (\mathbf {u}_h),\Psi (\mathbf {v})) =\langle \ell _h,\Psi (\mathbf {v})\rangle \quad \text {for all} \ \mathbf {v} \in \mathbb {R}^{m_h}. \end{aligned}$$

We note that, for any $\mathbf {u} \in \mathbb {R}^{m_h}$; $\mathsf {B}_h(\Psi (\mathbf {u}),\Psi (\cdot ),\Psi (\cdot )):\mathbb {R}^{m_h} \times \mathbb {R}^{m_h} \rightarrow \mathbb {R}$ is a symmetric and coercive bilinear form on $\mathbb {R}^{m_h} \times \mathbb {R}^{m_h}$ and thus can be represented by a symmetric positive definite matrix $\mathbf {A}_h^{\mu }(\mathbf {u}) \in \mathbb {R}^{m_h\times m_h}$ (which depends on $\mathbf {u} \in \mathbb {R}^{m_h}$). Likewise, $\langle \ell _h,\Psi (\cdot )\rangle :\mathbb {R}^{m_h} \rightarrow \mathbb {R}$ is a linear form and hence can be identified with a vector $\mathbf {b}_h \in \mathbb {R}^{m_h}$. Consequently, problem (25) can be restated as:

26 $$\begin{aligned} \text {find} \ \mathbf {u}_h \in \mathbb {R}^{m_h} \ \text {such that} \quad \mathbf {A}_h^{\mu }(\mathbf {u}_h) \cdot \mathbf {u}_h = \mathbf {b}_h; \end{aligned}$$

here and in the following, in the context of matrices and vectors in $\mathbb {R}^{m_h \times m_h}$ and $\mathbb {R}^{m_h}$, respectively, we denote by ’$\cdot$’ the usual matrix product.

### Algebraic gradient descent method and preconditioning

By invoking the isomorphism $\Psi : \mathbb {R}^{m_h} \rightarrow X_h$, the operator $\mathsf {F}_h:X_h \rightarrow X_h^\star$ from (24) can be considered as an operator $\mathbf {F}_h:\mathbb {R}^{m_h} \rightarrow \mathbb {R}^{m_h}$ given by

$$\begin{aligned} \mathbf {F}_h(\mathbf {u}):=\mathbf {A}_h^{\mu }(\mathbf {u}) \cdot \mathbf {u}- \mathbf {b}_h, \qquad \mathbf {u} \in \mathbb {R}^{m_h}. \end{aligned}$$

In particular, this is the algebraic gradient with respect to the Euclidean inner-product on $\mathbb {R}^{m_h}$ and the corresponding gradient descent method for (26) reads as

$$\begin{aligned} \mathbf {u}^{n+1}=\mathbf {u}^n-\delta (\mathbf {u}^n)\mathbf {F}_h(\mathbf {u}^n), \qquad n=0,1,2,\dotsc , \end{aligned}$$

where $\mathbf {u}^0 \in \mathbb {R}^{m_h}$ is an initial guess and $\delta (\mathbf {u}^n)>0$, for $n=0,1,2,\dotsc$, are a suitable step-sizes. We emphasise that the algebraic gradient is completely detached from the original partial differential equation (arising as the mathematical model of, e.g., a physical problem). Therefore, we should rather consider the discrete (vector) version of the generalised gradient from (13). In particular, for given $\mathbf {u} \in \mathbb {R}^{m_h}$, let $\mathbf {\nabla _{a_u} H(u)} \in \mathbb {R}^{m_h}$ be such that

$$\begin{aligned} a_{\Psi (\mathbf {u})}(\Psi (\mathbf {\nabla _{a_u} H(u)}),\Psi (\mathbf {v}))=\langle \mathsf {F}_h(\Psi (\mathbf {u})),\Psi (\mathbf {v})\rangle =\mathbf {v}^{T} \cdot \mathbf {F}_h(\mathbf {u}) \quad \text {for all} \ \mathbf {v} \in \mathbb {R}^{m_h}. \end{aligned}$$

Since $a_{\Psi (\mathbf {u})}(\Psi (\cdot ),\Psi (\cdot )):\mathbb {R}^{m_h} \times \mathbb {R}^{m_h} \rightarrow \mathbb {R}$ is a symmetric and coercive bilinear form, it can be represented by a symmetric positive definite matrix $\mathbf {P}_h(\mathbf {u})\in \mathbb {R}^{m_h \times m_h}$. Hence, we have that

$$\begin{aligned} \mathbf {\nabla _{a_u} H(u)}=\mathbf {P}_h(\mathbf {u})^{-1} \cdot \mathbf {F}_h(\mathbf {u}) \end{aligned}$$

and, in turn,

27 $$\begin{aligned} \mathbf {u}^{n+1}=\mathbf {u}^n-\delta (\mathbf {u}^n)\mathbf {P}_h(\mathbf {u}^n)^{-1} \cdot \mathbf {F}_h(\mathbf {u}^n), \qquad n=0,1,2,\dotsc . \end{aligned}$$

Especially, if $\mathbf {P}_h(\mathbf {u})=\mathbf {P}_h$ is independent of $\mathbf {u} \in \mathbf {R}^{m_h}$, then this procedure coincides with the algebraic gradient descent method for the preconditioned problem

28 $$\begin{aligned} \mathbf {P}_h^{-1} \cdot \mathbf {A}_h^{\mu }(\mathbf {u}_h) \cdot \mathbf {u}_h = \mathbf {P}_h^{-1} \cdot \mathbf {b}_h. \end{aligned}$$

In particular, in the light of our observations from Sect. 2, the algebraic preconditioner arises as the discretisation of the Sobolev preconditioner; or said differently, the preconditioned algebraic gradient method (27) matches, up to the damping parameter, the discretisation of the unified iteration scheme (6). We remark that this is not a new insight, but is already known in the literature: for linear problems, this and many more observations are well presented in [33], and in the context of nonlinear problems we refer to [24] and [9, Ch. 8].

We note that the approach above, i.e., to consider the discretisation of an operator preconditioner as the algebraic preconditioner, has some major advantages: First of all, this provides a natural way to choose an algebraic preconditioner and does not require any prior knowledge about the structure of the matrices. Furthermore, in the context of the interplay with a mesh refinement procedure, we simply have to adapt the discretisation of the operator preconditioner, which is straightforward. Most importantly, since the convergence of the (fixed-point) iteration schemes are established, in general, in the continuous setting, those results (most often) apply in the discrete setting as well and are independent of the mesh size; for the latter we further refer to [21, 22, 24], where the mesh independence of the condition number in the context of the Sobolev (gradient) preconditioners is verified in a rather general setting.

### Preconditioned nonlinear conjugate gradient method

As we have seen before, at least for our model problem, the gradient descent method with respect to an inner-product $a(\cdot ,\cdot )$, induced by a linear and invertible operator $\mathsf {P}:X \rightarrow X^\star$, simply leads to the preconditioned algebraic gradient descent method in the discretise setting, whereby the algebraic preconditioner is given by the discretisation of the operator $\mathsf {P}$. Consequently, if we want to derive the conjugate gradient method for the case that the gradient is taken with respect to some (variable) inner-product $a_u(\cdot ,\cdot )$, $u \in X$, on *X*, this simply leads to the known preconditioned nonlinear conjugate gradient method, see Algorithm 1. More details about (the derivation of) this method can be found, e.g., in the book [46], see also the article [6]. We further refer to [1] for a convergence analysis of the PNCG method.
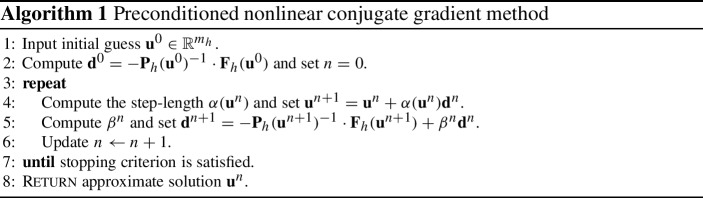


#### Remark 3.1

Without going into too much details, we shall provide some comments on Algorithm 1.

1. Ideally, the step-size $\alpha (\mathbf {u}^n)\ge 0$ is chosen so that
  29 $$\begin{aligned} \alpha (\mathbf {u}^n)={{\,\mathrm{arg\,min}\,}}_{\alpha \ge 0} \mathsf {H}(\Psi (\mathbf {u}^n+\alpha \mathbf {d}^n)). \end{aligned}$$
  In practice, however, this minimiser will only be approximated by using, e.g., a line search or a trusted region method; we refer, e.g., to [26, §3.2]. Often, especially for convergence proofs, it is required that the choice of the step-size satisfies some version of the Wolfe conditions. The standard Wolfe conditions were introduced in [54, 55]. Later on, several modified Wolfe conditions were presented; we refer to [15] and the references therein.
2. Many different choices for the conjugate gradient update parameter $\beta ^n$ have been proposed in the literature, see, e.g., the extensive survey of Hager and Zhang [15] and the references therein. For the PNCG method, two of the most popular choices are the ones proposed by Fletcher and Reeves [10],
  30 $$\begin{aligned} \beta _{FR}^n=\frac{\mathbf {F}_h(\mathbf {u}^{n+1})^{T} \cdot \mathbf {P}_h(\mathbf {u}^{n+1})^{-1}\cdot \mathbf {F}_h(\mathbf {u}^{n+1})}{\mathbf {F}_h(\mathbf {u}^{n})^{T} \cdot \mathbf {P}_h(\mathbf {u}^{n})^{-1} \cdot \mathbf {F}_h(\mathbf {u}^{n})}, \end{aligned}$$
  and by Polak and Ribière [43] and Polyak [44],
  $$\begin{aligned} \beta _{PR}^n=\frac{[\mathbf {F}_h(\mathbf {u}^{n+1})-\mathbf {F}_h(\mathbf {u}^{n})]^T \cdot \mathbf {P}_h(\mathbf {u}^{n+1})^{-1} \cdot \mathbf {F}_h(\mathbf {u}^{n+1})}{\mathbf {F}_h(\mathbf {u}^{n})^T \cdot \mathbf {P}_h(\mathbf {u}^{n})^{-1} \cdot \mathbf {F}_h(\mathbf {u}^{n})}. \end{aligned}$$
  Later on, Powell proposed in the article [45] the following modified (and improved) version of the parameter $\beta ^n_{PR}$:
  31 $$\begin{aligned} \beta _{PR+}^n=\max \{\beta _{PR}^n,0\}. \end{aligned}$$

## Numerical experiment

In this section, we run a numerical experiment to compare the performance of the various iteration schemes introduced in Sect. 3.1 and their conjugated counterparts. To this end we consider our model problem (17), where $\Omega :=(-1,1)^2 \setminus [0,1] \times [-1,0] \subset \mathbb {R}^2$ is an L-shaped domain and the diffusion coefficient $\mu$ obeys the Carreau law; i.e., we have that

32 $$\begin{aligned} \mu (t)=\mu _\infty +(\mu _0-\mu _\infty )(1+\lambda t)^{\nicefrac {(r-2)}{2}}, \end{aligned}$$

with $\mu _0> \mu _\infty >0$, $\lambda >0$, and $r \in (1,2)$. It is straightforward to verify that this choice of the diffusion coefficient satisfies (16) with $m_\mu =\mu _\infty$ and $M_\mu =\mu _0$. Moreover, since $r \in (1,2)$, the diffusion coefficient is decreasing. Therefore, the Zarantonello, Kačanov, and Newton methods converge for appropriate damping parameters. The source term $g \in L^2(\Omega )$ is chosen in such a way that the unique solution of (17) is given by the smooth function

$$\begin{aligned} u^\star (x,y)=\sin (\pi x)\sin (\pi y), \end{aligned}$$

where $(x,y) \in \mathbb {R}^2$ denote the Euclidean coordinates. Furthermore, for the discretisation of problem (17), we consider the conforming $\mathbb {P}_1$-finite element method, i.e., we set $p=1$ in (21), whereby the mesh $\mathcal {T}_h$ consists of 196,608 triangles. In our experiment below, we choose the parameters $\mu _\infty =1$, $\mu _0=100$, $\lambda =2$, and (a) $r=1.4$ or (b) $r=1.05$, respectively. In order to approximate the corresponding solutions of the discretised problem (22) for the parameters from (a) and (b), respectively, we will apply the Kačanov method with 1000 iteration steps. Subsequently, we will examine how many iteration steps are required by the Zarantonello, Kačanov, and Newton methods, as well as their conjugated counterparts with update parameters from (30) and (31), respectively, in order to obtain an error tolerance of $10^{-6}$ with respect to the norm $\left\| \cdot \right\| _X$ in *X*. In each case we choose the function $u^0 \equiv 0 \in X_h$ as our initial guess. Moreover, the one-dimensional optimisation problem from line 4 in the PNCG Algorithm 1, cf. (29), is solved by the Matlab subroutine *fmincon* from the optimisation toolbox with standard options; this part of the algorithm could certainly be improved by a more sophisticated minimisation procedure.

In Table 1 we record the number of iteration steps that were performed by our nonlinear solvers to obtain an error tolerance of $10^{-6}$. If this accuracy was not achieved within 100 iteration steps, then the calculations were aborted, signified by ’-’ in the table. The damping parameters for the Zarantonello iteration (18) were chosen to be $\delta _Z=0.01$ in (a) and $\delta _Z=0.02$ in (b), respectively, as they seemed to be close to optimal. Morever, in both cases we set $\delta _N \equiv 1$ in (19), i.e., we considered the classical (undamped) Newton method. We emphasise that neither the algebraic gradient descent nor the conjugate gradient method (without preconditioning) converged in a reasonable number of iteration steps; hence, they are not included in the table.

**Table 1:** The required number of iteration steps for the various nonlinear solvers to obtain an error tolerance of $10^{-6}$

	(a) $r=1.4$			(b) $r=1.05$		
	FP	$\beta _{FR}^n$	$\beta _{PR+}^n$	FP	$\beta _{FR}^n$	$\beta _{PR+}^n$
Zarantonello	61	15	15	–	36	36
Kačanov	25	9	10	90	19	24
Newton	5	7	6	7	15	8

Here, ’FP’ signifies the usual fixed-point iteration. If the prescribed accuracy was not obtained within 100 steps, then this is remarked by the symbol ’–’ in the table

**Fig. 1:**
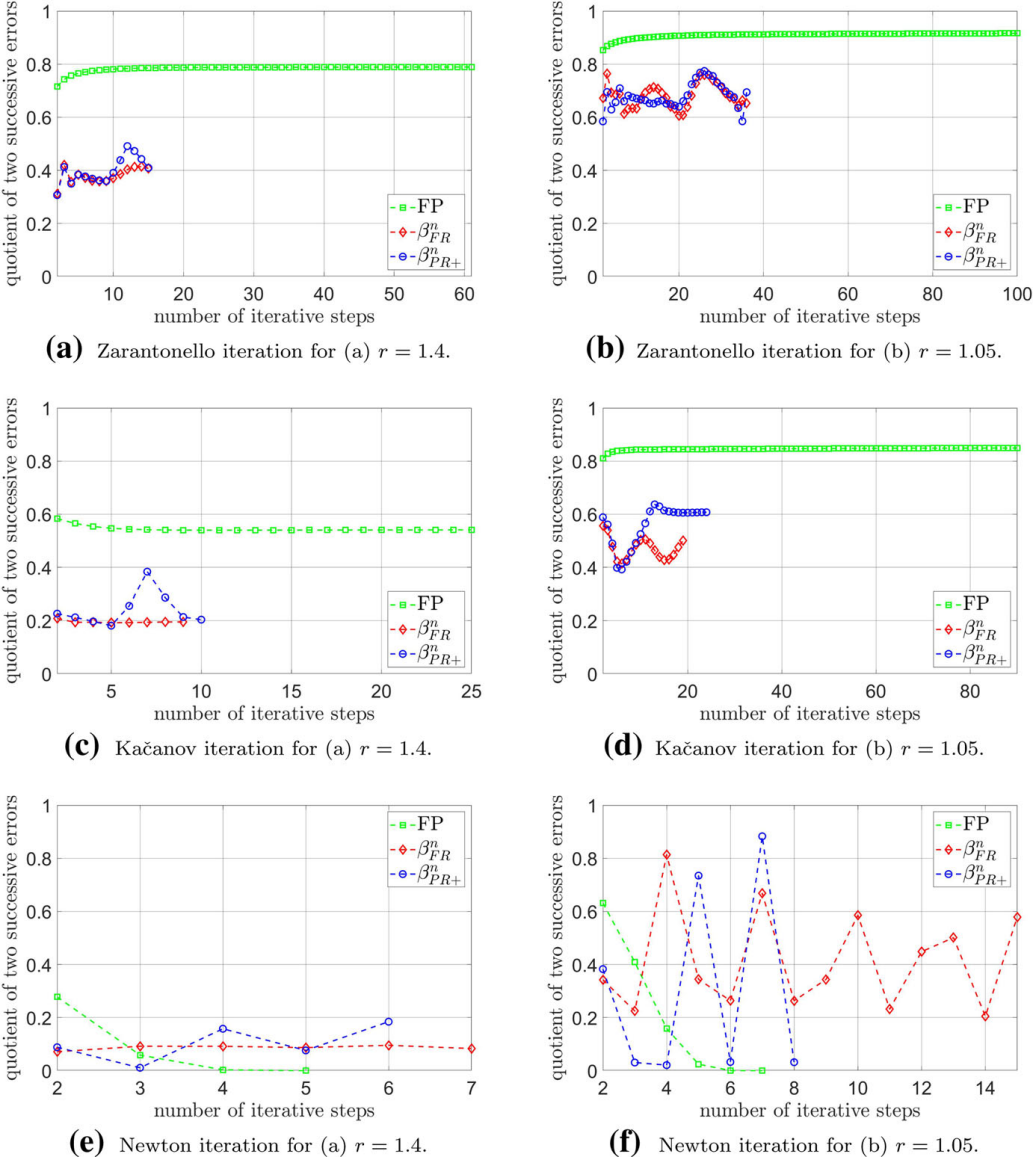
Plot of the quotient of two successive errors in the *X*-norm against the number of iterative steps

As we can see from Table 1, the Newton method outperformed the other iteration schemes in the specific problem considered. Indeed, the classical Newton method was even slightly superior to its conjugated counterparts; this was possibly caused by a suboptimal numerical solution of the one-dimensional optimisation problem (29) in our computations, whereas the classical Newton method exhibits quadratic convergence close to a solution. In contrast, considering the Kačanov and Zarantonello schemes, we observe that their corresponding PNCG methods require significantly less iterations to obtain the prescribed error tolerance, at least in the given experiment. Indeed, as can be seen in Fig. 1, the quotient of two successive errors, measured in the *X*-norm, are always smaller for the corresponding PNCG methods compared to the Zarantonello and Kačanov schemes, which, in contrast, is not the case for the Newton method. However, we should be aware that the preconditioned conjugate gradient methods require an additional solution of a one-dimensional minimisation problem, which, in general, is not for free.

**Table 2:** Comparison of the required number of iteration steps for different mesh sizes and the various nonlinear solvers to obtain an error tolerance of $10^{-6}$

	noe: 24,833			noe: 196,608			noe: 786,432		
	FP	$\beta _{FR}^n$	$\beta _{PR+}^n$	FP	$\beta _{FR}^n$	$\beta _{PR+}^n$	FP	$\beta _{FR}^n$	$\beta _{PR+}^n$
Zarantonello	61	16	16	61	15	15	61	15	16
Kačanov	25	9	10	25	9	10	25	9	10
Newton	5	7	7	5	7	6	5	7	6

Here, ”noe” is used as an abbrevation for ”number of elements”

It remains to experimentally highlight the mesh independence of our iterative solvers. For that purpose, we rerun our experiment (a) from before for both a coarser and a finer mesh; we remark that they are hierarchical meshes, i.e., the finer meshes are obtained by a (uniform) mesh refinement of the coarser meshes. As shown in Table 2, the number of iteration steps required to obtain an error tolerance of $10^{-6}$ is indeed independent of the mesh size, at least for the experiment considered herein.

Finally, we note that for more complicated problems the domain of convergence for the Newton scheme can be rather small, and thus we have to consider other nonlinear solvers such as the Kačanov and Zarantonello methods; see, e.g., [17, §5.1]. Hence, it is certainly worth to study those iteration schemes. Moreover, as we have observed above, their conjugated counterparts are able to accelerate the convergence (in view of the number of iteration steps), at least for the model problem considered.

## Conclusion

As we have seen, up to a damping function, the fixed-point iteration obtained by a preconditioning operator coincides with the steepest descent method using the variable inner-product that is induced by this preconditioning operator. Moreover, in view of the corresponding discretised problem in $\mathbb {R}^{m_h}$, the operator preconditioner acts as an algebraic preconditioner and, in turn, leads to the preconditioned algebraic gradient descent method. Our numerical experiment illustrated that the choice of a problem related (operator) preconditioner may significantly improve the convergence of the nonlinear conjugate gradient method. Furthermore, in that case, the convergence rate is independent of the mesh size.

## Footnotes

1 Also known as the Picard, simple, or Richardson iteration.

2 Further expressed as the secant modulus method or the frozen coefficient iteration.
